# Freiburg Neuropathology Case Conference: A 51-year-old Patient Presenting with Transient Speech Disorder and a Mass Lesion in the Right Parietal White Matter

**DOI:** 10.1007/s00062-022-01195-6

**Published:** 2022-07-26

**Authors:** N. F. Belachew, M. Diebold, P. C. Reinacher, M. Prinz, H. Urbach, D. Erny, C. A. Taschner

**Affiliations:** 1 Departments of Neuroradiology, University of Freiburg, Breisacherstraße 64, 79106 Freiburg, Germany; 2 Neuropathology, University of Freiburg, Freiburg, Germany; 3 Stereotactic & Functional Neurosurgery, University of Freiburg, Freiburg, Germany; 4 Medical Centre, Faculty of Medicine, University of Freiburg, Freiburg, Germany; 5 Fraunhofer Institute for Laser Technology, Aachen, Germany

**Keywords:** Primary CNS lymphoma, Primary vasculitis of the CNS, Invasive high-grade grade glioma, Neurosarcoidosis, Extraosseous myeloma, Cerebral metastasis

## Case Report

**Fig. 1:**
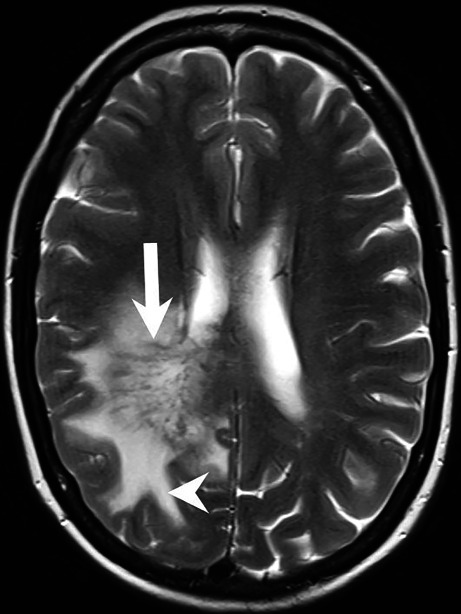
Axial T2-weighted images showed a lesion restricted to the white matter of the right frontoparietal lobe. The lesion consisted of hypointense linear structures radially connecting subependymal areas of the right lateral ventricle with the subcortical areas of the right frontal and parietal lobe (*arrow*). The lesion was surrounded by an extensive perifocal edema (*arrowhead*)

**Fig. 2:**
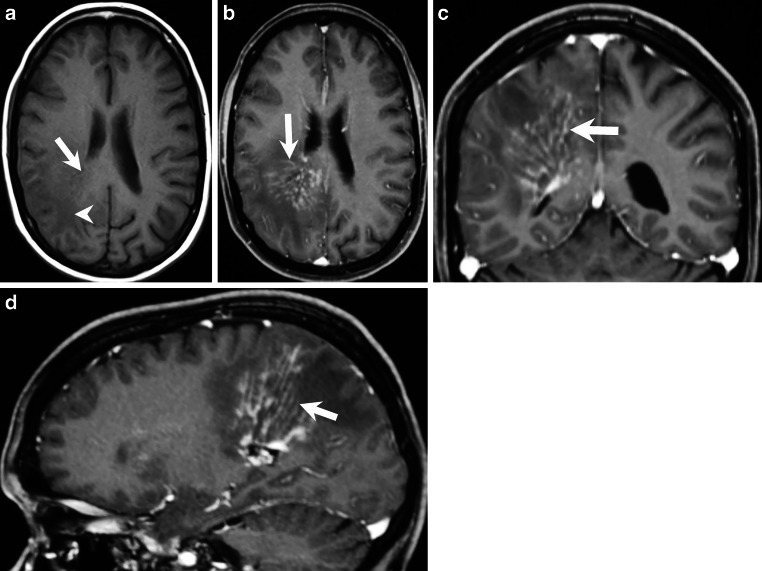
On axial, native T1-weighted images (**a**) the linear components of the lesion appeared hypointense (*arrow*) when compared to the cerebellar tissue with a distinct perifocal edema surrounding these structures (*arrowhead*). On axial (**b**), coronal (**c**), and sagittal (**d**) T1-weighted images after administration of gadolinium the linear structures show marked enhancement of contrast (**b**–**d**, *arrowhead*) most likely corresponding to perivascular spaces

**Fig. 3:**
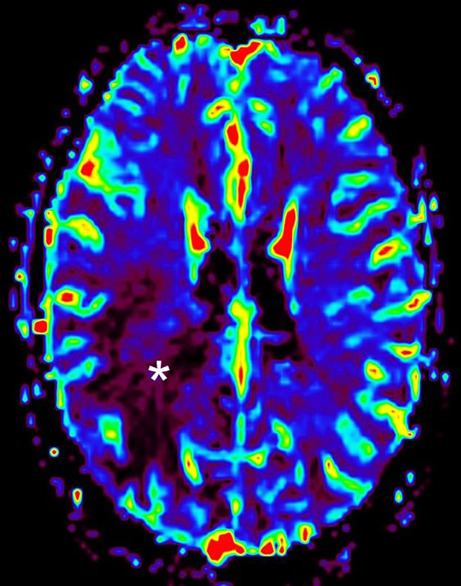
On axial parametric perfusion imaging maps representing the cerebral blood flow (relative cerebral blood volume, rCBV) the contrast-enhancing portions of the lesion display no hyperperfusion (*asterisk*)

The 51-year-old female patient presented to a peripheral hospital with new onset dizziness accompanied by brief episodes of speech disturbance. A computed tomography (CT) of the head was performed, which revealed a right parietal mass lesion (not shown). The patient was then transferred to our hospital. On admission, the patient was awake, oriented, without focal neurological deficits, no speech disorder, no headache. Magnetic resonance imaging (MRI) of the neurocranium revealed a space-occupying lesion in the right parietal white matter (Figs. 1, 2 and 3). The case was thoroughly discussed in our interdisciplinary brain tumor conference and the indications for stereotactic biopsy were established.

Stereotactic biopsy was performed the following day with the patient under general anesthesia after obtaining informed consent. The head was fixed in a Leksell stereotaxic frame (G-frame, Elekta, Stockholm, Sweden) and CT angiography was performed for planning. A serial biopsy was conducted in the right parietal paraventricular target region. A side-cutting biopsy needle was introduced via a spiral drill trephination using a right parietal approach. The neuropathologist present in the operating room confirmed that a pathologic process was biopsied.

The postoperative course was uneventful, the patient had no new focal neurological deficits and could be discharged on postoperative day 3. Further treatment will be performed in the hemato-oncology department.

## Imaging

The cranial MRI upon admission (Figs. 1, 2 and 3) revealed a space-occupying lesion restricted to the white matter of the right parietal lobe. The lesion consisted of hypointense linear structures radially connecting subependymal areas of the right lateral ventricle with the subcortical areas of the right frontal and parietal lobe (Figs. 1 and 2a, arrow). On T1-weighted images after administration of gadolinium (Fig. 2b–d, arrow) these linear structures displayed homogeneous contrast enhancement most likely representing perivascular structures. The lesion was surrounded by an extensive perifocal edema disproportional to the size of the underlying lesion (Figs. 1 and 2a, arrowhead). On parametric perfusion imaging maps (relative cerebral blood volume, rCBV) the lesion was not hyperperfused (Fig. 3, asterisk). Diffusion weighted images (DWI) with a b-value = 1000 showed no signs of restricted diffusion within the lesion (not shown).

## Differential Diagnosis

### Primary Central Nervous System (CNS) Lymphoma

Primary central nervous system lymphomas (PCNSL) are relatively rare (accounting for only 4% of all intracranial neoplasms) with a peak incidence at about 60 years [1, 2]. The World Health Organization (WHO) classification of tumors has defined a number of subtypes according to the presumed ethology and genesis [3].

In contrast to the imaging presented in this case report, lymphomas typically present as a hyperdense mass on NCCT with vivid and homogeneous enhancement after contrast administration and restricted diffusion on DWI [4]. While a solitary manifestation can be observed in the majority of cases, PCNSL may present as multiple intra-axial lesions in about 30–40% of patients [4]; however, just as in the case presented PCNLS have a predilection for the periventricular white matter and may show no or irregular enhancement mimicking primary angiitis of the CNS [5]. The frequency of simultaneous diagnosis of PCNSL and primary vasculitis of the CNS observed, suggests an immunologic paraneoplastic mechanism [6].

### Primary Vasculitis of the CNS

Primary vasculitis of the CNS (PVCNS) is an extremely rare disorder with an estimated average annual incidence of 2.4 cases per 1 million of the population [7]. While it may affect patients of all ages and both genders, the highest risk is found among males around 50 years of age [8]. The clinical and radiological diagnosis remains challenging as symptoms and imaging may show a variety of nonspecific findings.

Ischemic manifestations as observed in the left hemisphere of the patient presented in this case report are the most common [7]; however, they usually affect multiple vascular territories in both hemispheres [7]. In the majority of cases PVCNS also cause intracranial haemorrhage, parenchymal, subarachnoid or both [7]. A tumor-like presentation is found in about 1 of 10 patients [7]. While the most characteristic enhancement pattern presents with very small partial rims on axial sections, some patients may show variable patterns including filiform perivascular enhancement similarly to the one seen in Figs. 1 and 2b–d [7]. PVCNs usually also leads to the steno-occlusive vascular lesions involving proximal or distal segments of at least two cerebral arteries [7]. There was no specific vascular imaging performed in the initial imaging work-up of the case presented here that would have allowed us to rule out occlusive disease within the intracranial arteries.

### Invasive High-grade Glioma

Due to their highly invasive nature, malignant gliomas (i.e. astrocytomas IDH mutant grade 3 or 4 and glioblastomas IDH wildtype grade 4 were considered in this case) tend to spread along subcortical white matter and intrahemispheric as well as interhemispheric tracts, such as the corona radiata and the corpus callosum [9]. While high-grade IDH mutant astrocytomas mainly affect adults between 30 and 50 years of age [10], the peak incidence for glioblastomas is around 55 years [11]. As in this case, malignant gliomas are usually solitary, hypointense to isointense on T1, hyperintense T2/Fluid attenuated inversion recovery (FLAIR) and predominantly affect the white matter [12]. Although contrast enhancement may be variable, it is most often seen peripherally and surrounding necrotic portions of the tumor [12]; however, invasion of the perivascular spaces has been reported [9] and may lead to a more unusual enhancement pattern. Perifocal vasogenic oedema is seen in most of the larger gliomas but is usually less extensive as seen in the case presented. The solid and enhancing parts of the lesion may show restricted diffusion and an elevated relative cerebral blood volume (rCBV) which tend to correlate with portions within the tumor that are more likely to be high grade [13].

### Neurosarcoidosis

Neural involvement is relatively common among patients suffering from systemic sarcoidosis [14]. The high number of intracranial findings associated with neurosarcoidosis and their subtle manifestation in some cases may pose a challenge in patients who are neither known nor suspected to suffer from sarcoidosis prior to brain imaging. Sarcoidosis usually affects patients between 30 and 40 years of age [15]. An MRI may show enhancement of different intracranial structures including the brain parenchyma (in the form of masses or nodules), the pachymeninges and leptomeninges as well as the cranial nerves [16]. Leptomeningeal enhancement predominantly involves the basal aspects of the brain but may also follow perforating vessels along perivascular spaces leading to a distinct enhancement pattern as seen in this Figs. 1 and 2b–d [17]. Cranial nerve involvement most commonly affects the facial and the optic nerve and may occur in up to three quarters of patients [18, 19]. Pituitary and hypothalamic involvement are usually seen in cases with more extensive leptomeningeal involvement but may also present in isolation [16].

### Cerebral Metastasis

Due to the great variation in imaging appearances brain metastasis present a well-known diagnostic challenge. They roughly represent one third of all intracranial tumors and are most often caused by either lung carcinoma, colorectal carcinoma or breast carcinoma [20]. Cerebral metastasis may be occult at diagnosis and can be solitary in up to 50% of cases [20]. They predominantly occur at the grey-white matter junction or in the arterial watershed areas and may hemorrhage [21]. They may be of variable density on NCCT and often show surrounding edema that is disproportional to the tumor size [21]. On MRI they are usually isointense to hypointense on T1, hyperintense on T2/FLAIR and may show intense, punctate, nodular or ring enhancement depending on their size [21]; however, case reports have shown that brain metastasis can also lead to enhancement along perivascular spaces [22] as seen in this case. Thus, it is wise to consider metastasis as a potential differential diagnosis in all patients with an intra-axial mass lesion, regardless of the enhancement pattern.

### Extramedullary Plasmacytoma

Extramedullary plasmactyomas (EMPC) are solitary plasmacytomas manifesting as isolated accumulations of plasma cells at non-osseous sites. They account for less than 5% of all plasma cell disorders and up to one third of solitary plasmacytomas [23]. Patients affected usually present between the fifth and seventh decade of life with a localized submucosal mass in the head and neck region involving either the sinuses, the nasopharynx or the oropharynx [23].

It has also been reported that there is a relevant male predilection [23]. Cross-sectional imaging usually shows homogeneously enhancing soft tissue masses [24]. While literature review provides a few cases describing intracranial plasmocytoma, they have all been reported as extra-axial masses in patients suffering from multiple myeloma [25–27]. To our knowledge, this is the first case report presenting a patient with an intra-axial solitary plasmacytoma that shows no connection to the dura or skull interface on cross-sectional imaging. Considering the location and the imaging characteristics of the tumor, biopsy and histopathological analyses were essential for making the correct final diagnosis.

## Histology and Molecular Pathology

**Fig. 4:**
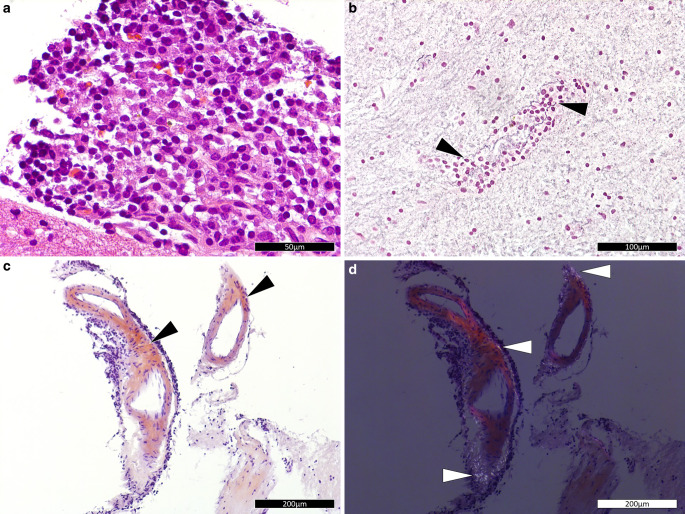
Staining of stereotactic biopsy. Hematoxylin and eosin stained section (**a**) showing a lymphoplasmacytic infiltrate in the lesion. (Size bar: 50 µm) Tibor Papanicolaou (**b**) stained section with evidence of splinted basal lamina indicated by arrow heads. (Size bar: 100 µm) Congo red stained section (**c**) in conventional brightfield mode with red deposits (*arrowheads*). Identical Congo red stained section in polarized light mode (**d**) with bottle green deposits (*arrowheads*), **c** and **d** illustrate amyloid deposition, a common feature of extraosseous plasmacytomas. (Size bars **c** and **d**: 200 µm)

A stereotactic biopsy collected fragments of brain parenchyma and tissue with increased cellularity from the region of the abovedescribed lesion. In the hematoxylin and eosin (H&E) stained sections of the formaldehyde-fixed and paraffin-embedded initial biopsy material, the dense areas emerged as lymphoplasmacytic infiltrates with predominant plasma cell morphology (Fig. 4a). Silver staining revealed splitting of the basal lamina in resident vessels with the plasma cell infiltrates amidst endothelial cells (Fig. 4b). Amyloid depositions were identified in the lesion by Congo red stain (Fig. 4c,d). The lymphoplasmacytic infiltrates appeared to be predominantly in perivascular regions while only single lymphoplasmacytic cells were found in the brain parenchyma in this small biopsy. Adjacent regions exhibited slightly increased cell density and astrogliosis, but no evidence of necrosis, demyelination or axonal destruction (not shown).

**Fig. 5:**
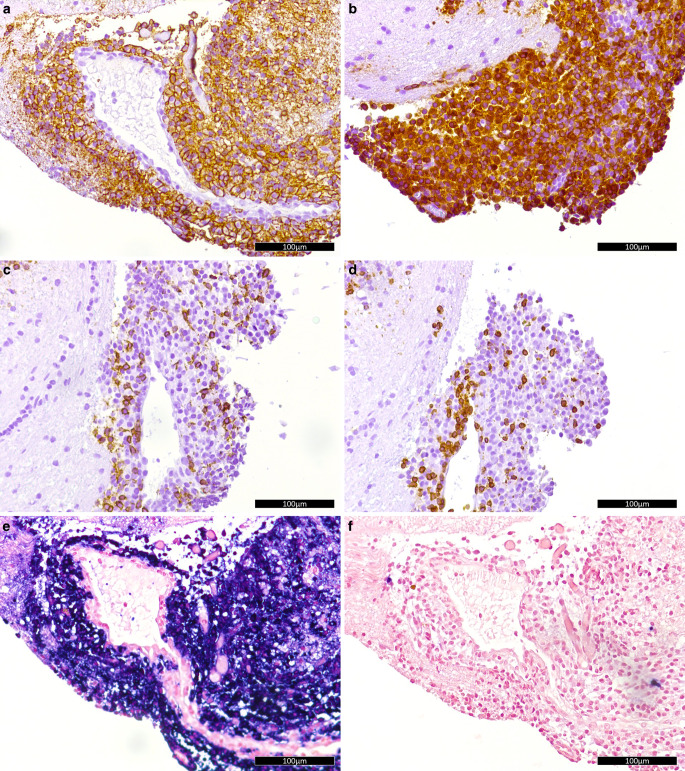
Immunohistochemistry and in-situ hybridization. Immunohistochemistry of the lesion for markers: CD138 (**a**), CD79a (**b**), CD20 (**c**) and CD3 (**d**); brown color indicates marker expression. In situ hybridization with probes for kappa (**e**) and lambda (**f**) chains; dark purple signal indicates positive signal. (All size bars: 100 µm)

The infiltrate was dominated by moderately large lymphoplasmacytic cells with abundant cytoplasm and an eccentric nucleus. Besides their morphology these cells were identified as plasma cells by their immunohistochemical positivity for CD38 and CD138 (Fig. 5a). These cells further strongly expressed CD79a (Fig. 5b). Circulating B cells with CD20 expression (Fig. 5c), T cells with CD3 expression (Fig. 5d), and macrophages with CD68 expression were only loosely scattered within the lesion. The lymphoplasmacytic infiltrate showed a slightly increased proliferative activity (~1%) as assessed by ubiquitin-protein ligase MIB1 (not shown).

Immunohistochemically, a kappa/lambda disbalance with kappa restriction was observed in the plasma cells and confirmed by in situ hybridization (Fig. 5e,f). Fragment length analysis of IgH FR I and FR II further documented a reproducible rearrangement. Finally, panel sequencing was performed with proof of wildtype sequences for C‑X‑C chemokine receptor type 4 (CXCR4) and myeloid differentiation primary response protein 88 (MYD88) in the lesion.

### Diagnosis

#### Extraosseous Plasmacytoma

Histomorphology and clonality identify this lesion as a plasma cell neoplasm. The spectrum of plasma cell neoplasms comprises monoclonal gammopathy of undetermined significance (MGUS), plasma cell myeloma, as well as rarer plasmacytoma variants like an extraosseous plasmacytoma [28]. The specific diagnosis is based on a combination of the immune phenotype, molecular pathology, and the clinical work-up. In this case the hematologic diagnostics including laboratory and radiological assessment as well as a bone marrow biopsy did not yield evidence of an extracranial manifestation of the neoplasm, up to the date of publication. In conclusion, an extraosseous plasmacytoma was postulated.

These solitary extramedullary plasma cell neoplasms have mainly been described in the upper aerodigestive tract (approx. 80% of cases), but also have been described to occur in other locations, including the gastrointestinal and urinary tract, breasts, thyroid glands, testes, parotid glands, skin, and CNS [28–31]. In the present case, a single manifestation in the cerebrum and no serum monoclonal protein were observed, as complies with the diagnostic criteria [28, 32]. A male preponderance and an average age of 55 years at manifestation have been reported in the literature [28, 32]. Only 10–15% of cases progress to manifest plasma cell myeloma, according to case series [28, 32]. Compared to plasma cell myeloma with extramedullary spread, the solitary extramedullary plasmacytoma was reported to have a better prognosis with a 25% local recurrence rate after radiotherapy [28, 32].

The initial differential diagnosis comprised lymphomas, vasculitides as well as other infectious or autoimmune vessel-associated CNS disorders. The highly plasma cell-dominated histomorphology with relatively scarce T and B cell infiltration and without appearance of necrotic areas, however, led to the conclusion of a neoplastic event, early in the diagnostic process. No suggestive features of the more common variants of CNS-associated hematolymphoid tumors (specifically a primary diffuse/intravascular large B‑cell lymphoma) mentioned in the WHO classification of CNS tumors [3] were observed; however, the diagnosis of a lymphoplasmacytic lymphoma of the CNS, known as Bing-Neel syndrome, was considered [28, 33]. The absence of IgM paraprotein and of a typical MYD88 L265P mutation made this diagnosis improbable.

In conclusion, this rare case of a solitary extraosseous plasmacytoma of the CNS illustrates the importance of a concerted diagnostic approach between neuroradiological interpretation, brain biopsy with molecular pathological diagnostics and hematologic counselling and work-up.
